# Supplementary material for: Stability Studies of Clonazepam 2.5 mg/mL Oral Solution and 1 mg/mL Parenteral Solution in Pre-Filled Polypropylene Syringes
Source: Pharmaceutics. 2025 Oct 7;17(10):1302. doi: 10.3390/pharmaceutics17101302 (PMC12567341; doi:10.3390/pharmaceutics17101302)
Supplement: Supplementary file 1 [file pharmaceutics-17-01302-s001.zip › pharmaceutics-3857192-supplementary.pdf]

Supplementary Table ST1: Peak area and the clonazepam concentration for making calibration curve.

Point	Concentration (μg/mL)	Peak area (μV*sec)
1	0	1.05E+03
1	0	7.53E+02
1	0	9.67E+02
1	0	9.14E+02
1	0	9.02E+02
2	6	7.32E+05
2	6	7.21E+05
2	6	7.22E+05
2	6	7.26E+05
2	6	7.36E+05
3	15	1.98E+06
3	15	1.95E+06
3	15	1.97E+06
3	15	1.94E+06
3	15	1.96E+06
4	24	3.09E+06
4	24	3.08E+06
4	24	3.10E+06
4	24	3.09E+06
4	24	3.11E+06
5	30	3.85E+06
5	30	3.85E+06
5	30	3.88E+06
5	30	3.88E+06
5	30	3.86E+06
6	45	5.71E+06
6	45	5.70E+06
6	45	5.67E+06
6	45	5.67E+06
6	45	5.77E+06

Supplementary Figure SF1: Linearity between the peak area and the clonazepam concentration for making calibration curve.

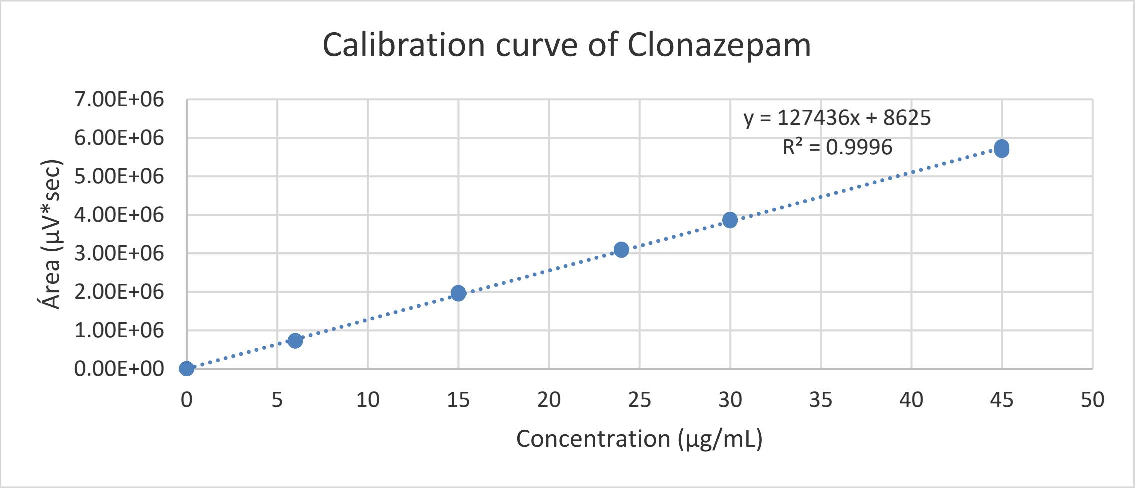

Supplementary Table ST2: Data from intra-day repeatability of clonazepam samples at 80, 100 and 120% of the target concentration of 25 µg/mL

Target concentration (µg/mL)	Estimated concentration (µg/mL)	Mean	SD	RSD%	RSD% mean	Accuracy%	Accuracy% mean
20 (80%)	20.031	20.039	0.029	0.147	0.308	100.156	100.196
	20.009					100.045	
	20.029					100.144	
	20.088					100.440	
	20.039					100.193	
25 (100%)	25.020	24.994	0.024	0.094		100.079	99.977
	24.985					99.941	
	25.014					100.055	
	24.961					99.845	
	24.992					99.968	
30 (120%)	29.870	30.035	0.205	0.682		99.567	100.116
	29.905					99.684	
	29.898					99.660	
	30.329					101.096	
	30.172					100.574	

SD= Standard deviation; RSD%: Relative standard deviation

Supplementary Table ST3: Data from inter-day repeatability of clonazepam samples at 80, 100 and 120% of the target concentration of 25 µg/mL

Target concentration (µg/mL)	Estimated concentration (µg/mL)	Day	Mean	SD	RSD (%)	RSD% mean	Accuracy %	Accuracy% mean
20 (80%)	19.931	1	*20.042*	*0.066*	*0.331*	0.425	99.656	100.212
	20.134	1					100.672	
	20.073	2					100.365	
	20.036	2					100.178	
	19.977	3					99.875	
	20.109	3					100.547	
	20.029	4					100.147	
	20.049	4					100.243	
25 (100%)	25.037	1	25.037	0.058	0.232		100.147	100.149
	24.982	1					99.927	
	25.111	2					100.442	
	24.968	2					99.871	
	25.102	3					100.409	
	24.981	3					99.924	
	25.027	4					100.109	
	25.091	4					100.362	
30 (120%)	30.326	1	30.096	0.214	0.713		101.086	100.319
	29.919	1					99.730	
	29.879	2					99.595	
	30.287	2					100.956	
	29.895	3					99.649	
	30.195	3					100.649	
	29.909	4					99.695	
	30.358	4					101.194	

SD= Standard deviation; RSD%: Relative standard deviation

Supplementary Table ST4: Data from accuracy of clonazepam samples at 80, 100 and 120% of the target concentration of 25 µg/mL (recovery expressed as a percentage of recovery of theorical value -100%-)

Theorical concentration (µg/mL)	Estimated concentration (µg/mL)	Recovery%	Mean	SD	RSD%
20	19.412	97.060%	99.984	2.516	2.517
20	19.487	97.435%			
20	19.456	97.280%			
25	25.006	100.024%			
25	24.806	99.224%			
25	24.958	99.832%			
30	30.994	103.313%			
30	30.962	103.207%			
30	30.744	102.480%			

Recovery%= Percentage of recovery of theorical value (100%)

Supplementary Table ST5: Average Recovery% of three determinations on the day of the test and pH of oral clonazepam 2,5 mg/mL solution in pre-filled syringes at room temperature

Test Day	C1 (µg/mL)	Recovery% of concentration C1	C2 (µg/mL)	Recovery% of concentration C2	C3 (µg/mL)	Recovery% of concentration C3	Average Recovery% of concentration	pH
0	25.06	100.24%	24.99	99.96%	24.95	99.80%	100.00%	4.63
1	24.87	99.49%	25.01	100.02%	25.08	100.31%	99.94%	4.53
2	25.23	100.91%	25.10	100.40%	25.23	100.93%	100.75%	4.59
3	25.13	100.53%	25.13	100.53%	25.35	101.39%	100.82%	4.62
4	25.18	100.72%	25.39	101.57%	25.32	101.30%	101.19%	4.63
7	25.44	101.75%	25.46	101.83%	25.43	101.72%	101.77%	4.63
9	25.75	102.99%	25.77	103.09%	25.70	102.80%	102.96%	4.64
11	25.52	102.08%	25.59	102.34%	25.54	102.18%	102.20%	4.66
14	25.10	100.41%	25.28	101.11%	25.24	100.97%	100.83%	4.67
17	25.30	101.19%	25.39	101.56%	25.36	101.45%	101.40%	4.67
21	25.72	102.89%	25.75	102.98%	25.58	102.30%	102.73%	4.68
24	25.05	100.18%	25.01	100.04%	25.02	100.07%	100.10%	4.71
30	25.29	101.18%	25.15	100.58%	24.81	99.24%	100.33%	4.77

Supplementary Table ST6: Average Recovery% of three determinations on the day of the test and pH of oral clonazepam 2,5 mg/mL solution in pre-filled syringes under refrigerated conditions

Test Day	C1 (µg/mL)	Recovery% of concentration C1	C2 (µg/mL)	Recovery% of concentration C2	C3 (µg/mL)	Recovery% of concentration C3	Average Recovery% of concentration	pH
**0**	25.06	100.24%	24.95	99.80%	25.00	100.00%	100.01%	4.65
**1**	25.08	100.32%	25.13	100.54%	25.08	100.30%	100.39%	4.55
**2**	24.77	99.07%	24.77	99.07%	24.75	99.00%	99.05%	4.60
**3**	25.06	100.22%	25.13	100.54%	25.09	100.35%	100.37%	4.63
**4**	24.05	96.20%	24.20	96.79%	24.18	96.73%	96.58%	4.63
**7**	24.90	99.59%	24.78	99.11%	24.79	99.18%	99.29%	4.64
**9**	25.12	100.47%	24.72	98.90%	25.01	100.05%	99.81%	4.65
**11**	25.26	101.03%	25.29	101.16%	25.31	101.23%	101.14%	4.65
**14**	24.58	98.31%	24.30	97.19%	24.43	97.71%	97.74%	4.67
**17**	25.06	100.25%	25.28	101.13%	25.18	100.72%	100.70%	4.67
**21**	25.30	101.21%	25.22	100.89%	25.29	101.14%	101.08%	4.68
**24**	24.28	97.11%	23.98	95.91%	24.24	96.98%	96.67%	4.70
**30**	24.92	99.68%	23.98	95.93%	24.47	97.87%	97.82%	4.75

Supplementary Table ST7: Average Recovery% of three determinations on the day of the test and pH of parenteral clonazepam 1 mg/mL solution in pre-filled syringes at room temperature

Test Day	C1 (µg/mL)	Recovery% of concentration C1	C2 (µg/mL)	Recovery% of concentration C2	C3 (µg/mL)	Recovery% of concentration C3	Average Recovery% of concentration	pH
0	25.10	100.42%	25.06	100.23%	25.01	100.02%	100.22%	4.15
1	25.00	100.00%	24.88	99.53%	25.12	100.49%	100.00%	4.03
2	25.84	103.36%	25.54	102.15%	25.36	101.44%	102.31%	4.08
3	24.51	98.06%	24.52	98.09%	24.49	97.97%	98.04%	4.14
4	24.89	99.57%	24.93	99.71%	24.72	98.87%	99.38%	4.25
7	24.64	98.56%	24.65	98.61%	24.74	98.98%	98.72%	4.25
9	24.53	98.10%	24.85	99.41%	24.85	99.41%	98.97%	4.25
11	24.71	98.84%	25.26	101.06%	25.14	100.54%	100.15%	4.25
14	25.06	100.22%	25.22	100.88%	25.27	101.09%	100.73%	4.25
17	25.38	101.53%	25.55	102.21%	25.41	101.63%	101.79%	4.36
21	25.18	100.73%	25.27	101.06%	25.07	100.26%	100.69%	4.42
24	25.55	102.19%	26.08	104.33%	26.06	104.24%	103.59%	4.47
30	25.09	100.36%	25.17	100.69%	25.39	101.57%	100.87%	4.53

Supplementary Table ST8: Average Recovery% of three determinations on the day of the test and pH of parenteral clonazepam 1 mg/mL solution in pre-filled syringes at room temperatura protected from light

Test Day	C1 (µg/mL)	Recovery% of concentration C1	C2 (µg/mL)	Recovery% of concentration C2	C3 (µg/mL)	Recovery% of concentration C3	Average Recovery% of concentration	pH
0	25.10	100.42%	25.06	100.23%	25.01	100.02%	100.22%	4.15
1	24.57	98.29%	24.64	98.57%	24.73	98.92%	98.59%	4.19
2	25.35	101.39%	25.33	101.32%	25.24	100.98%	101.23%	4.21
3	24.08	96.31%	24.30	97.20%	24.09	96.36%	96.62%	4.23
4	24.65	98.60%	24.89	99.57%	24.66	98.64%	98.94%	4.26
7	24.78	99.14%	24.65	98.60%	24.78	99.12%	98.95%	4.28
9	24.65	98.61%	24.97	99.88%	25.58	102.32%	100.27%	4.29
11	25.03	100.11%	24.65	98.62%	24.11	96.45%	98.39%	4.3
14	24.50	98.01%	24.43	97.74%	24.66	98.64%	98.13%	4.32
17	24.95	99.81%	24.84	99.36%	24.66	98.65%	99.27%	4.34
21	25.48	101.93%	25.71	102.85%	25.50	102.01%	102.26%	4.37
24	25.13	100.51%	24.88	99.53%	25.27	101.09%	100.38%	4.39
30	24.59	98.34%	24.89	99.57%	24.13	96.51%	98.14%	4.41

Supplementary Table ST9: Average Recovery% of concentration and pH of parenteral clonazepam 1 mg/mL solution in pre-filled syringes under refrigeration conditions protected fom light

Test Day	C1 (μg/mL)	Recovery% of concentration C1	C2 (μg/mL)	Recovery% of concentration C2	C3 (μg/mL)	Recovery% of concentration C3	Average Recovery% of concentration	pH
0	25.10	100.42%	25.06	100.23%	25.01	100.02%	100.22%	4.15
1	24.68	98.70%	25.31	101.26%	25.61	102.44%	100.80%	4.1
2	25.53	102.10%	25.55	102.20%	25.45	101.80%	102.03%	4.12
3	24.36	97.43%	24.51	98.03%	24.37	97.47%	97.64%	4.13
4	24.79	99.16%	24.93	99.72%	24.95	99.79%	99.56%	4.14
7	25.25	100.99%	25.19	100.74%	25.44	101.74%	101.16%	4.15
9	25.14	100.57%	25.18	100.71%	24.97	99.87%	100.38%	4.16
11	25.73	102.93%	25.70	102.81%	25.72	102.89%	102.88%	4.17
14	25.60	102.41%	25.68	102.70%	25.42	101.67%	102.26%	4.18
17	24.98	99.90%	25.20	100.81%	24.95	99.80%	100.17%	4.19
21	25.66	102.63%	25.64	102.57%	25.62	102.46%	102.55%	4.2
24	25.52	102.09%	25.37	101.46%	24.51	98.04%	100.53%	4.22
30	24.88	99.53%	24.73	98.90%	23.91	95.63%	98.02%	4.23
